# Ephedrae Herba: A Review of Its Phytochemistry, Pharmacology, Clinical Application, and Alkaloid Toxicity

**DOI:** 10.3390/molecules28020663

**Published:** 2023-01-09

**Authors:** Songyuan Tang, Junling Ren, Ling Kong, Guangli Yan, Chang Liu, Ying Han, Hui Sun, Xi-Jun Wang

**Affiliations:** 1 National Chinmedomics Research Center, National TCM Key Laboratory of Serum Pharmacochemistry, Metabolomics Laboratory, Department of Pharmaceutical Analysis, Heilongjiang University of Chinese Medicine, Heping Road 24, Harbin 150040, China; 2 State Key Laboratory of Quality Research in Chinese Medicine, Macau University of Science and Technology, Avenida Wai Long, Taipa, Macao 999078, China

**Keywords:** Ephedrae Herba, phytochemistry, pharmacological effect, clinical application, alkaloid toxicity

## Abstract

Ephedrae Herba (*Ephedra*), known as “MaHuang” in China, is the dried straw stem that is associated with the lung and urinary bladder meridians. At present, more than 60 species of *Ephedra* plants have been identified, which contain more than 100 compounds, including alkaloids, flavonoids, tannins, sugars, and organic phenolic acids. This herb has long been used to treat asthma, liver disease, skin disease, and other diseases, and has shown unique efficacy in the treatment of COVID-19 infection. Because alkaloids are the main components causing toxicity, the safety of *Ephedra* must be considered. However, the nonalkaloid components of *Ephedra* can be effectively used to replace ephedrine extracts to treat some diseases, and reasonable use can ensure the safety of *Ephedra*. We reviewed the phytochemistry, pharmacology, clinical application, and alkaloid toxicity of *Ephedra*, and describe prospects for its future development to facilitate the development of *Ephedra*.

## 1. Introduction

**Figure 1:**
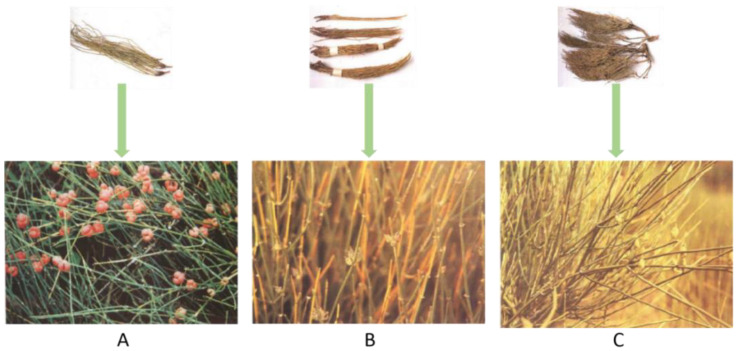
Three types of *Ephedra* listed in the 2020 edition of the *Pharmacopoeia of the People’s Republic of China*: (**A**) *Ephedra sinica* Stapf; (**B**) *Ephedra intermedia* Schrenk et C. A. Mey; (**C**) *Ephedra equisetina* Bge.

Ephedrae Herba (*Ephedra*), also known as “MaHuang” in China, grows mostly in dry desert environments and has been used in traditional Chinese medicine for more than 5000 years [1]. In the 2020 edition of the *Pharmacopoeia of the People’s Republic of China*, *Ephedra* include the dried straw stems of *Ephedra sinica* Stapf, *Ephedra intermedia* Schrenk et C. A. Mey., or *Ephedra equisetina* Bge (Figure 1A–C). The whole plant is acrid, slightly bitter, warm in nature, and associated with the lung and bladder meridians. It is often used in the clinical treatment of asthma, increasing blood pressure, and analgesia. Moreover, *Ephedra* can also be used to treat COVID-19 infections to improve the symptoms. Many beneficial components in *Ephedra*, including alkaloids, flavonoids, polysaccharides, and so on, have unique pharmacological effects. However, the mechanism of its efficacy is not completely explained in the literature, and the corresponding molecular structure has not been fully described and summarized. In addition, the U.S. Food and Drug Administration (FDA) announced in 2004 that additional dietary supplements cannot be used with *Ephedra*, and *Ephedra* use in other countries is also restricted [2]. The main reason for this is mainly due to the *Ephedra* alkaloids having certain toxicity (dose-related), although evidence shows that the nonalkaloid components in *Ephedra* are still capable of treating diseases. However, its clinical safety is still unknown, and the toxicity of *Ephedra* has not been thoroughly discussed. In this review, we present an overall overview of the phytochemical properties, pharmacological activities, clinical application, and alkaloid toxicity of *Ephedra*. By analyzing and integrating the study results, we describe the practical value of *Ephedra* and propose possible research future directions. We outline the shortcomings of the existing studies, thereby providing a valuable compilation and analysis of the current research on *Ephedra* plants and laying a foundation for the future Ephedra research.

## 2. Phytochemistry

Ephedra has a complex chemical composition and contains various types of compounds, including alkaloids, flavonoids, tannins, polysaccharides, and organic phenolic acids. Modern medical studies have shown that Ephedra has a wide range of pharmacological effects on the central nervous system, cardiovascular system, and smooth muscle [3,4,5]. Its active constituents mostly include Ephedra alkaloids, and the nonalkaloid components exhibit antioxidant, immunosuppressive, and hypoglycemic properties [6,7].

### 2.1. Alkaloids

Alkaloids are the main active components of Ephedra, in which a total of 29 have been identified (Table 1). The contents of the following three pairs of stereoisomeric amphetamine alkaloids are the highest: L-ephedrine and D-pseudoephedrine, L-norephedrine and D-norpseudoephedrine, and L-methylephedrine and D-methylpseudoephedrine. These three pairs of stereoisomers are generally considered to be the active ingredients of Ephedra (collectively referred to as ephedrines). Many methods can be used to determine the content of ephedrine alkaloids [8,9,10]. Ibragic et al. established a method for the rapid determination of ephedrine and pseudoephedrine content within 7 min using UPLC-UV, and measured the total content of ephedrine at a dry weight of 20.8 mg/g and pseudoephedrine at a dry weight of 34.7 mg/g. These two compounds accounted for 90% and 99% of the total alkaloid content, respectively [11]. In addition, the alkaloids in Ephedra include imidazole, amphetamine-type, quinoline, and pyrrolidine alkaloids, among others (Table 1, Figure S1).

### 2.2. Flavonoids

Flavonoids generally refer to a series of compounds in which two benzene rings (A and B rings) with phenolic hydroxyl groups are connected to each other through the three central carbon atoms. The total flavone content (TFC) extracted from Ephedra by ethanol in a water bath can be analyzed by HPLC/PDA/MS, and is approximately 0.29% [33,34]. At present, more than 40 kinds of flavonoids, such as quercetin, luteolin, and rutin, have been isolated from Ephedra. The results of pharmacological studies have shown that flavonoids in Ephedra can scavenge diphenylpicrohydrazide free radicals, and the strength of its antioxidant effect is related to the number and structure of hydroxyl groups of its active ingredients [35] (Table 2, Figure S2).

### 2.3. Tannins

The tannins make up a class of polyphenolic compounds with complex structures that are widely found in plants. They usually exist in condensed form in Ephedra, including dimer, trimer, and tetramer proanthocyanidins, as well as hydrolytic tannins (Table 3, Figure S3). The latest method for detecting tannins in Ephedra is gel permeation chromatography, developed by a team from Japan [52].

### 2.4. Polysaccharides

Polysaccharides are macromolecular components in Ephedra plants. At present, the main polysaccharides isolated from Ephedra are polysaccharides A, B, C, D, and E, and hyperbranched acidic polysaccharides (ESP-B4). Among them, the relative molecular masses of Ephedra polysaccharides A, B, C, D, and E are 1.2 × 10^6^, 1.5 × 10^6^, 9 × 10^4^, 6.6 × 10^3^, and 3.4 × 10^4^, respectively. In addition, when Ephedra stems were continuously extracted by the water extraction method (liquid–solid ratio 5) in a water bath at 90 °C for 3 h, the monosaccharide composition of the water-soluble polysaccharides in Ephedra was obtained as 43.1% glucose, 36.4% galactose, 14.9% mannose, 3.7% arabinose, and 1.7% gluconic acid [57].

### 2.5. Organic Acids

Cottiglia et al. separated phenolic acids, such as nebrodenside A, nebrodenside B, and O-coumaric acid glucoside, from *Ephedra* for the first time in 2005 [43]. Since then, scientists have also successively separated organic acids such as trans-cinnamic acid and syringin from Ephedra (Table 4, Figure S4).

### 2.6. Organic Volatile Essential Oil

The volatile oil in Ephedra is one of its medicinal material bases [63,64]. The content of volatile oil in Ephedra is low, at only approximately 0.15% [65]. Different planting methods, processing methods, and extraction techniques affect the content of volatile oil in Ephedra [66,67,68]. We provide a summary of the 30 common volatile oils in Ephedra in Table 5 and Figure S5.

### 2.7. Other Ingredients

In addition to the above compounds isolated and identified from Ephedra, other ingredients such as lignans, naphthalenes, esters, terpenoids, and quinones have been identified (Table 6, Figure S6).

## 3. Pharmacological Effects

### 3.1. Antipyretic and Diaphoretic Effects

The ephedrine, volatile oil, and their decoctions in Ephedra all produce different degrees of antipyretic and diaphoretic effects. The diaphoretic effect is especially notable. Wang et al. investigated the heat-inducing mechanism of Ephedra by adopting UPLC-Q/TOF-MS, and discovered that the 2-adrenoceptor (β2-AR) is a signal channel that modulates human perspiration [74]. Jo et al. randomly divided patients into an ephedrine injection group and normal saline injection control group and observed that esophageal temperature and hemodynamic variables remained stable in the patients injected with ephedrine, whereas those in the control group injected with normal saline substantially decreased. Compared with the control group, the index finger temperature of the patients in the ephedrine group was markedly lower. This showed that ephedrine can minimize the core temperature, maintain the overall temperature, and stabilize the maintenance of hemodynamic variables [75]. Carlisle et al. studied the effect of intraperitoneal ephedrine on the metabolic rate of rats in cold (−8 °C) and thermoneutral (22 °C) environments. They found that the diaphoretic effect of ephedrine is closely related to the ambient temperature: the higher the ambient temperature, the better the diaphoretic and heat-generating effect [76]. This also further confirmed that the mechanism of Ephedra diaphoretic may be closely related to the function of the central nervous system [77]. In addition, other researchers have found that Ephedra can reduce the IL-1β level and inhibit HSP70 and NF-κB to promote hypothalamic homeostasis and inhibit heat stress [78]. To summarize, Ephedra plays antipyretic and sweating roles mainly by inhibiting the release of fever factors and controlling the perception by the central nervous system of environmental temperature, but the specific pathway still needs further exploration.

### 3.2. Inhibiting Asthma

**Figure 2:**
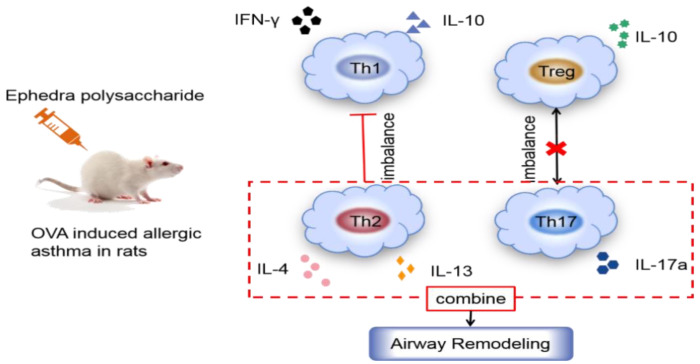
*Ephedra* polysaccharide regulates OVA-induced inflammatory response in asthma.

In traditional Chinese medicine, Ephedra is thought to stimulate warmth, relax the qi machine, open the skin, and unblock the qi in the lungs; because of its bitterness, the lungs are internally rejuvenated, which calms coughing. Mei et al. randomly divided Wistar rats into five groups, including a control group and an aspirin group, and the groups received three different doses of ephedrine plaster (6, 12, or 24 g/kg). The antiasthma effect of ephedrine plaster was evaluated with an ovalbumin (OVA)-induced asthma rat model. The fever caused by inflammation in the rats in the ephedrine plaster groups was significantly reduced in a dose-dependent manner; the increase in eosinophils caused by OVA was also reduced, and the lung dry weight ratio in the antiasthma test significantly decreased [79]. These findings indicated that the Ephedra-gypsum extract had antipyretic and antiasthmatic effects. Ma et al. established a mouse asthma model induced by OVA, used an enzyme-linked immunosorbent assay (ELISA) to assess Th1/Th2 and Th17 cytokine levels, and assessed Th17 cell flow cytometry with an ELISA assay to determine the antiasthmatic effect of a Mahuang decoction (consisting of Ephedra, Glycyrrhizae Radix et Rhizoma, Cinnamomi Ramulus, and Armeniacae Semen Amarum), with *Ephedra* being the key ingredient. The results showed that the Mahuang decoction could inhibit Th17 cells, and effectively inhibited the progression of asthma, which indicated that the Mahuang decoction could be used as a therapeutic drug for patients with allergic asthma [80]. Recently, ELISA, quantitative real-time reverse transcription (qRT)-PCR, and flow cytometry (FCM) were used to measure the levels of inflammatory factors and inflammatory cells in asthmatic model rats, which showed that Ephedra polysaccharides could improve the symptoms of asthmatic rats by regulating the immune imbalance in Th1/Th2 and Th17/Treg cells [81]; the specific pathway is shown in Figure 2. Huang et al. used selective and rapid HPLC/MS-MS to study the ephedrine, pseudoephedrine, methylephedrine, and other ephedrines in an asthma rat model established by the OVA sensitization method after using a Mahuang decoction. The blood concentration of the main components of the decoction showed that it could control or improve asthma, with a lag between the peak drug concentration and the maximum therapeutic effect [82]. Tang et al. found that the Mahuang decoction can be taken together with theophylline to treat cough and asthma [83]. In addition, aerosol administration of Ephedra aqueous extract could inhibit asthma, because it inhibited IL-13 and eotaxin protein expression, reduced airway inflammation, and controlled eosinophil infiltration [84].

### 3.3. Anti-Inflammatory Effect

**Figure 3:**
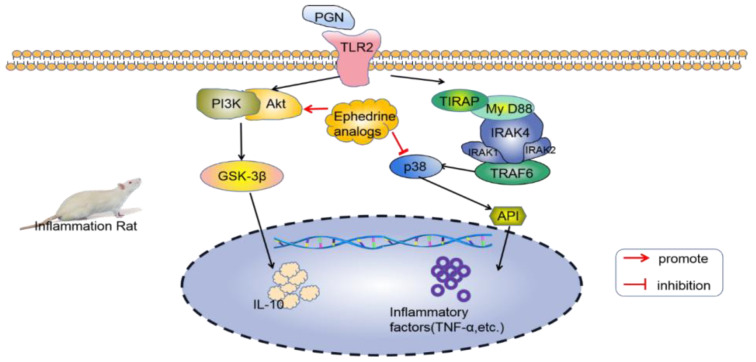
Mechanism through which *Ephedra* inhibits inflammatory factors in mice with peritonitis.

Ephedra contains anti-inflammatory chemicals. In 1985, ephedrine, pseudoephedrine, and ephedrine analogs were found to have strong anti-inflammatory activities in vivo. Its anti-inflammatory effect may be achieved by inhibiting the biosynthesis of prostaglandin E2 [85]. Ephedra protected mice from endotoxin injury by inhibiting the expression of inflammatory factors induced by Gram-positive bacteria (G+), which was due to the Toll-like receptor 2 (TLR2) stimulation by peptidoglycan (PGN) in macrophages. Ephedrine administration can produce an anti-inflammatory effect by stimulating the expression of IL-10 in dendritic cells (DCs) and inhibiting the production of induced inflammatory factors (such as TNF-α) through the PI3K/Akt and PGN pathways. Moreover, ephedrine can act on PI3K, Akt, and the downstream GSK-3β and p38 pathways, leading to the increased expression of IL-10 and the decreased expression of inhibitory inflammatory factors [86,87]; the specific mechanism is summarized in Figure 3.

He et al. found that Ephedra had a strong inhibitory effect on the secretion of inflammatory mediators and inflammatory cell infiltration in the lung tissue of asthmatic rats; it inhibited the expressions of IL-21, IL-21R, STAT3, and p-STAT3 in lung tissue [88]. In addition, pseudoephedrine has anti-inflammatory effects. Wu et al. used high-performance liquid chromatography with a photodiode array detector (HPLC-PDA) and utilized the fingerprint screening of anti-inflammatory components to identify the characteristics and active components of Suhuangzhenke capsules (SHs) as quality control markers. The results showed that pseudoephedrine significantly inhibited inflammatory mediator NO production in LPS-stimulated RAW264.7 macrophages, which proved that pseudoephedrine also has anti-inflammatory effects [89]. Zhai et al. found that Ephedra could substantially reduce the degree of alveolitis and pulmonary fibrosis, and had a therapeutic effect on idiopathic pulmonary fibrosis rats induced by bleomycin [90,91]. When *Ephedra* plays an anti-inflammatory role, many of the active substances or receptors are the same or belong to the same family as those that are active when relieving asthma, fever, and sweating. Therefore, effective substances in *Ephedra* may simultaneously play different roles through the same pathway, which provides ideas for further understanding the role of some of the pathways.

### 3.4. Hepatoprotection Effect

In 1984, Japanese researchers successfully isolated feruloyl histamine from Ephedra and found that it has an inhibitory effect on histidine decarboxylase and antihepatotoxicity [15]. Furthermore, Han et al. found that the alkaloids in Ephedra protected the livers of rats in a model of D-galactosamine–lipopolysaccharide (D-GalN/LPS)-induced acute liver failure by inhibiting the high expression of tumor necrosis factor [92]. Ghasemi et al. determined the hepatoprotective effect of an Ephedra extraction on carbon tetrachloride (CCl4)-induced chronic and acute liver failure mouse models, and the antioxidant activity of Ephedra extraction was determined by 2,2′-diphenyl-1-pyridohydrazine (DPPH) and β-carotene bleaching methods. The findings revealed that after treatment with an Ephedra extraction, all parameters of liver inflammation significantly decreased in liver injury mice, which could be attributed to the hepatoprotective effects of Ephedra, which functioned to inhibit oxidative stress and reduce liver inflammation [93]. Ephedra significantly inhibited the apoptosis of hepatocytes and could thus treat acute liver failure by inhibiting the activities of D-galactosamine (GalN) and lipopolysaccharide (LPS). This thereby inhibited the activities of serum alanine aminotransferase (ALT); total bilirubin (T Bil); and caspases 8, 9, and 3, resulting in curbing liver failure. The authors speculated that Ephedra may increase the level of transcription activating factor 3 (STAT3) by increasing the level of IL-6, which may be an effective therapeutic pathway through which Ephedra treats acute liver failure [94]. Song et al. found that an ephedrine alkaloid-free Ephedra extraction regulated lipid metabolism by inhibiting the production of free radicals and helping with the recovery of liver function [95]. The above studies show that the active ingredients in Ephedra can protect the liver (mainly due to its antioxidative and antiapoptotic properties), inhibit the secretion of inflammatory factors, as well as regulate liver lipid metabolism to produce a unique hepatoprotective effect. Ephedra is more distinctive in traditional Chinese medicine. According to ancient books, it is effective in treating jaundice, although the foreign literature claims that it is harmful to the liver [96,97]; therefore, additional investigation is necessary regarding the suitably of the application of Ephedra for the liver.

### 3.5. Antibacterial and Antifungal Effect

The stems and seeds of *Ephedra* contain many antibacterial components [98,99], and the inhibitory effects and mechanisms of Ephedra on different types of bacteria are different. Phenolic compounds isolated from Ephedra have remarkable antibacterial activity against Gram-negative and -positive bacteria and fungi [99]. Ali et al. reported that three types of wild Ephedra (Ephedra Strobilacea, Ephedra Pachyclada, and Ephedra Procera) and their respective callus cultures showed strong antibacterial activities, but the original plants exhibited higher antibacterial activity against damaged tissues [100]. Zang et al. tested the antibacterial activity of 12 kinds of A-type proanthocyanidins isolated from Ephedra and found that these compounds inhibited Gram-positive and -negative bacteria and fungi at concentrations ranging from 0.00515 to 1.38 mmol·L^−1^ [44]. Similarly, the 4-quinolone-2-carboxylic acid isolated from Ephedra showed strong antibacterial activity against common bacteria such as Escherichia coli, Pseudomonas aeruginosa, and Staphylococcus aureus [23]. An Ephedra extraction also produced an excellent inhibitory effect on Aspergillus flavus and Aflatoxin. Researchers evaluated the effect of methanol extracts of the aerial parts and roots of Ephedra on the growth of Aspergillus parasiticus NRRL 2999 and the Aflatoxin B(1) (AFB(1)) produced by itself; they found that the concentration of the methanol extract positively correlated with the inhibition of fungal growth and the production rate of AFB(1). The IC50 and effective concentration values of AFB(1) were 559.74 and 3.98 μg·mL^−1^, respectively. The methanol extract also inhibited the growth of fungi [101]. All the extracts described above in Ephedra that play an antibacterial role are not Ephedra alkaloids, which means that Ephedra does not rely much on alkaloid components when it plays a specific role, which has reference value for subsequent studies on the safety of Ephedra drug use.

### 3.6. Anticancer and Analgesia

Although Ephedra is suitable for treating sweating and fever, it is also commonly used for pain relief in clinics. A study reported that a flavonoid aglycone present in herbacetin 7-*O*-neohsperidin in Ephedra has anticancer cell proliferation effects and analgesic properties [102]. It could also inhibit the abnormal proliferation of cancer cells induced by the hepatocyte growth factor (HGF) by inhibiting C-Met phosphorylation and its tyrosine kinase activity through the PI3K/Akt pathway [103,104]. Moreover, the expressions of tyrosinase genes, as well as B16F10 melanoma cells, were significantly dose-dependently inhibited by ephedrine A and B [105]. In another study, the remaining components in the aqueous phase, after the methanol extract of Ephedra was re-extracted with butanol and ethyl acetate, were able to significantly grow tumor blocks in BDF1 mice (30 mg/kg/d) [106]. Chinese herbal formulae incorporating Ephedra have been investigated primarily for the treatment of lung cancer, breast cancer, thyroid tumor, and other tumors. According to the above findings, Ephedra can be used for the treatment of clinical cancer, but in-depth research on the molecular mechanisms through which Ephedra functions in the treatment of various cancers has not yet been conducted, which may affect the subsequent clinical application of Ephedra.

### 3.7. Antivirus Effect

According to the *Compendium of Materia Medica·Cao Bu*, Ephedra ranked first among the drugs used for the treatment of heat toxic skin syndrome and Qi-Fen syndrome. Modern studies have also shown that Ephedra has a strong antiviral effect. Japanese scientists found that an Ephedra extract can induce the nuclear translocation of nuclear factor-κB (NF-κB) to eliminate the latent virus reservoir in people with human immunodeficiency virus type 1 (HIV-1) [107]. Additionally, Chinese scientists found that a Mahuang decoction could increase the levels of IL-2 and interferon-γ in mice by inhibiting the TLR4/MyD88/TRAF6 signaling pathway, and reduce the level of IL-4 and the expression of NF-κB, thus producing antiviral effects on mice infected with influenza virus (IFV) [108]. According to the prediction of network pharmacology, Mahuang decoctions may act on AKT1, TNF, TP53, IL6, JUN, and other targets through the PI3K/Akt, MAPK, and JAKSTAT signaling pathways, so as to prevent and treat influenza A [71].

## 4. Clinical Application

### 4.1. Treatment of Coronavirus Disease 2019 (COVID-19)

On 11 February 2020, the World Health Organization officially designated COVID-19 as a pandemic, which has spread around the world [109]. Traditional Chinese medicine showed a strong COVID-19 prevention and treatment effect, and a series of prescriptions showed therapeutic effects, such as Shufeng Jiedu capsules, a Maxing Shigan decoction, Lianhua Qingwen capsules/granules, a Qingfei Paidu decoction (QFPDT), etc. Among them, the QFPDT, which consists of 21 herbs, such as Ephedra, gypsum, and licorice, produced effective results across more than 10 provinces and cities in China. According to the study, the formula may effectively slow disease advancements in mild cases, decrease the duration of common and severe symptoms, and shorten hospital stay durations [110]. Yao et al. divided 42 patients with COVID-19 into a treatment group and a control group to estimate the therapeutic effect of Lianhua Qingwen granules; the control group received only basic treatment (Western medicine treatment). The results showed that compared with the control group, the fever symptoms of 18 patients in the treatment group decreased, accounting for 85.7% of all patients, which was significantly better than the 57.1% achieved in the control group (*p* < 0.05). Cough symptoms disappeared in seven cases (46.7%), which was significantly better than the 5.6% achieved in the control group (*p* = 0.012). Furthermore, the therapeutic impact of the treatment group was statistically superior to that of the control group in terms of reducing muscular pain, runny nose, headache, vomiting, and a lack of appetite [111]. In addition, the drug pair containing Ephedra played a key role in the treatment of COVID-19 [112,113]. Ang et al. found that when Ephedra was paired with gypsum fiber, it was effective in the treatment of pediatric COVID-19 [114]. The latest study showed that quinoline-2-carboxylic acid in Ephedra could effectively antagonize the active compounds produced by the interaction between angiotensin-converting enzyme 2 (ACE2) and SARS-CoV-2 spike protein receiver binding domain (SARS-CoV-2 RBD) and inhibit the viral infection, thus showing the potential of using Ephedra to treat COVID-19 infection [115]. This provides strong evidence that Ephedra is an effective medicine used for the treatment of COVID-19 infections. Through network pharmacological prediction, the therapeutic targets of Ephedra are likely TNF-α, IL2, FOS, ALB, and PTGS2 in the pathways related to respiration, nerves, blood circulation, and digestion [112].

Additionally, Ephedra is a pharmaceutical substance frequently used in the therapeutic treatment of COVID-19. Wang et al. reviewed the prescriptions of traditional Chinese medicine for patients with severe COVID-19, and they performed correlation analysis on drugs according to the principles of prescription analysis. They finally identified 1532 effective prescriptions; Ephedra was one of the high-frequency prescriptions. In the prescription analysis by Fan et al., Ephedra was also used up to 142 times. The above findings showed that Ephedra plays an important role in the treatment of COVID-19 infection [116,117]. These data fully demonstrate that Ephedra plays an important role in the treatment of COVID-19 infection, and provides inspiration for the future direction of the traditional Chinese medicine (TCM) treatment of disease.

### 4.2. Treatment of Asthma

Asthma is the most common chronic respiratory disease in the world and is associated with a persistent inflammatory response caused by a variety of immune cells. Ephedra, which has multiple targets and multiple function pathways, can effectively be used for the treatment of asthma. According to historical ancient books, in ancient times, Ephedra was used in combination with other drugs to treat cough and asthma in east Asia [118]. The ephedrine, pseudoephedrine, and volatile oils contained in Ephedra have antiasthmatic effects, among which ephedrine is the most powerful. Hou et al. found that in Qingfei Xiaoyan pills, the nonselective β-AR agonist ephedrine was the main bronchodilator, and ephedrine could synergize with lignins, such as arctiin, arctigenin, descurainoside, and descurainolide B, to produce a bronchodilation effect, and to treat cough and asthma [119]. Ephedra can be used in the clinic to treat asthma because it can regulate the immune imbalance in Th1/Th2 and Th17/Treg cells.

### 4.3. Raising Blood Pressure and Treating Muscle Weakness

In many circumstances, the blood pressure of the human body rapidly drops, such as during a major bleeding accident during an operation, in pregnant women, or in those who suddenly change their position, especially middle-aged and elderly people. This situation can sometimes be life-threatening. Muscle weakness is a disease of neuromuscular junction transmission dysfunction, which is clinically characterized by skeletal muscle fatigue. The common challenge in treating the two disorders is how to rapidly and forcefully develop muscular strength in order to raise blood pressure and alleviate muscle weakness. Because of the effect of ephedrine in stimulating the secretion of adrenaline and in increasing muscle strength, ephedrine is commonly used clinically to rescue hypotension and myasthenia gravis [120]. Santana et al. retrospectively evaluated several patients with idiopathic scoliosis whose mean arterial pressure dropped to below 60 mmHg during an operation and found a substantial association between blood pressure elevation and ephedrine consumption [121]. When Kitaura et al. intravenously injected 4 mg of ephedrine into patients with Parkinson’s disease whose blood pressure was significantly reduced, they observed unexpectedly large contractions and increased blood pressure from 78 to 168 mmHg, and the heart rate increased from 52 beats per minute (bpm) to 84 bpm. This phenomenon occurred every time when 4 mg of ephedrine was injected, showing that ephedrine has an effect on blood pressure [122]. Wang compared the efficacy and safety of the drugs (norepinephrine, phenylephrine, and ephedrine) used for hypotension in women with pre-eclampsia under spinal anesthesia during cesarean section. The results showed that the heart rate increase caused by norepinephrine and phenylephrine was significantly lower than that of ephedrine (80.5 ± 12 vs. 84.9 ± 7.1 bpm; *p* = 0.02), and fewer episodes of ephedrine tachycardia occurred, indicating that ephedrine not only quickly raised blood pressure, but was also safer [123]. The pressure-boosting mechanism of ephedrine may involve increasing the heart rate, and enhancing the myocardial contractility and cardiac output, thus increasing systemic and pulmonary blood pressure levels and the vasoconstriction response, which can then produce a positive inotropic effect, thus increasing the blood pressure [124]. In addition, a Xiaoxuming decoction containing Ephedra was used to effectively treat patients with myasthenia gravis and recurrent myasthenia gravis [125].

### 4.4. Analgesic Effect

Ephedra can produce an analgesic effect through the pseudoephedrine and polysaccharide contained in Ephedra, which can relax smooth muscle and relieve blood stasis [126]. Schachtel et al. conducted analgesia tests on 640 patients with upper respiratory tract infection through acetylsalicylic acid (ASA) and pseudoephedrine (PSE), and found that the analgesic effect and tolerance were good [127]. Basu found that the administration of oral pseudoephedrine in adults reduced the pain and trauma in the middle ear during aviation flight [128]. Yoshimura et al. reported that Ephedra extract components, other than Ephedra alkaloids, could inhibit cellular mesenchymal-to-epithelial transition factor (c-Met) to relieve pain [52]. The analgesic effect of Ephedra is presently thought to be produced by pseudoephedrine and polysaccharides in Ephedra, but the mechanism through which the effect is produced is not yet clear.

### 4.5. Treatment of Skin Diseases

Skin allergy is an allergic reaction, manifested as erythema, papules, and itching. Ephedra extract has antibacterial activity, which can prevent the invasion of microorganisms, thereby promoting wound healing. Many ancient Chinese medicine prescriptions for the treatment of skin diseases are related to Ephedra, such as Mahuang Lianqiao Chixiaodou decoctions for the treatment of dermatitis, Maxing Shigan decoctions for the treatment of skin allergies, etc. [129]. In all of these, Ephedra is the main drug. Although Ephedra is frequently applied in skin diseases, studies on its target and molecular mechanism are lacking, which will seriously affect the promotion of the clinical use of Ephedra.

### 4.6. Treatment of Gynecological Diseases

In traditional Chinese medicine, gynecological diseases are mostly considered syndromes of yang deficiency and cold coagulation. Ephedra can regulate yang, which can not only warm the meridians, but also invigorate qi and dispel cold. Adams et al. found that in California, many herbs including Ephedra are widely used to treat female dysmenorrhea and premenstrual syndrome [130]. Jaradat et al. also found that Ephedra is the most commonly used plant for breast cancer treatment in Palestine [131]. In addition, researchers used XTT analysis (tetrazolium salt reduction) to detect the cytotoxicity of ovarian cancer cell lines (A2780 and A2780CisR) and noncancerous kidney cells (HEK-293) after using Ephedra, and found that Ephedra treatment reduced sensitivity to cisplatin and the cytotoxicity of ovarian cancer cells [132].

## 5. Alkaloid Toxicity

The misuse and abuse of products containing Ephedra and its extracts have led to many toxic events over the past few decades. As of 2004, the U.S. Food and Drug Administration had received more than 18,000 reports of toxic reactions related to Ephedra [133], and banned the sale of dietary supplements containing Ephedra [2]. Toxic reactions when using Ephedra include excitement, sweating, dysuria, and increased blood pressure, as well as more severe cases including arrhythmia, nephritis, gallstones, and possibly death due to heart or respiratory failure [134,135,136]. Scientists found that a high dose (1000 mg/kg) of Ephedra aqueous extract significantly increased the number of basophils in renal tubules, the weight of salivary glands, and the hypertrophy acinar cells of both female and male F344 rats [137]. When ephedrine and caffeine were used together, F344 rats showed muscle fiber degeneration or even loss [138]. In another group of animal experiments, high concentrations of Ephedra or ephedrine (dosage equivalent to 12.5 to 50 mg/kg of ephedrine) caused cardiac toxicity in rats, which positively correlated with the dosage [139].

The cause of the adverse reactions to Ephedra is its alkaloids; thus, scientists have analyzed the safety of Ephedra extracts without Ephedra alkaloids. Takemoto et al. used ephedra extract (EHE) and ephedra extract without ephedra alkaloids (EFE) to test the activity time, forced swimming time, and pentobarbital-induced sleep time of mice in an open field. They found that both could increase the activity of mice in the open field, reduce the immobility time in forced swimming, and reduce the sleep time in sleep tests. However, compared with mice in the EHE group, the mice in the EFE group did not have arrhythmia, suggesting that EFE may be used as a substitute for EHE without side effects in the future [7]. Kobayashi also found that EHE without Ephedra alkaloids not only suppressed pain, but also did not have the side effects of Ephedra alkaloids such as sleep deprivation and arrhythmia [140]. Both EHE and EFE showed similar analgesic effects after oral administration, whereas EFE did not show the side effects of EHE [126]. In addition, it was reported that two groups of subjects were injected with EHE and EFE respectively for treatment, and the incidence of adverse events in the EHE treatment group was higher than that in the EFE group, but it was not statistically significant [141]. In addition, EFE preserved the anticancer properties of Ephedra, which indicated its developmental potential as a new therapeutic drug [142]. More studies are indicating that the nonalkaloid components in Ephedra can equally replace Ephedra extracts to treat some diseases without producing the side effects related to the latter. This suggests that further clinical studies are needed to determine the safety and efficacy of the nonalkaloid components of Ephedra. Currently available information shows no serious adverse events, and Ephedra is not listed as a poisonous medicinal material in the Chinese Pharmacopoeia.

However, prescription is a commonly used form of administration in Chinese medicine; that is, Ephedra is rarely used alone for treatment, but is instead used in combination with other drugs. Therefore, the safety of the prescription containing Ephedra must be known. When the concentration is 100 times higher than the adult clinical dosage, that is, a body weight of 23,000 mg/kg, a Mahuang Dingchuan decoction (consisting of Ephedra, Ginkgo Semen, Farfarae Flos, Pinelliae Rhizoma, Mori Cortex, Perillae Fructus, Scutellariae Radix, and Glycyrrhizae Radix et Rhizoma) had no obvious acute toxicity to mice and was relatively safe [143]. However, the mice died 1 h after the high-dose administration of a Mahuang decoction (consisting of Ephedra, Cinnamomi Ramulus, Armeniacae Semen Amarum, and Glycyrrhizae Radix et Rhizoma), with an LD50 of 51.07 g/kg [144]. Therefore, attention should be paid to the dosage when using any prescription containing Ephedra in clinical practice.

## 6. Conclusions and Future Perspective

We reviewed the botany, chemical composition, pharmacological action, clinical application, and toxicity of *Ephedra*. At present, more than 60 species of *Ephedra* have been identified, which can be mainly divided into *Ephedra sinica* Stapf, *Ephedra intermediate* Schrenk et C. A. Mey, and *Ephedra equisetina* Bge, which contain more than 100 compounds including alkaloids, flavonoids, tannins, sugars, and organic phenolic acids. The pharmacological effects that have been identified include antipyretic, antiasthmatic, anti-inflammatory, and liver protective effects, among others. Its clinical applications are extensive, as it can be used to treat asthma, liver disease, skin disease, and COVID-19 infection. However, its toxicity cannot be ignored. *Ephedra* alkaloids represent the main cause of toxic reactions, but evidence shows that *Ephedra* extracts without alkaloids have similar efficacy in treating some diseases with no adverse reactions.

Although *Ephedra* has a long been widely applied, some aspects require further study. First, more than 20 medicinal plants of *Ephedra* have been studied for their pharmacological activities, but the chemical constituents and pharmacological activities of many other *Ephedra* plants have not been studied. Therefore, to expand upon the applications of *Ephedra*, these untested species must be examined. Second, the mechanisms through which the many components of *Ephedra* treat some diseases remain unclear; for example, the effects of *Ephedra* polysaccharides on hyperlipidemia and its immunosuppression mechanism are still unclear, as are the effective substances and mechanisms of action of *Ephedra* in producing antibacterial properties. Therefore, further studies of its molecular mechanism, pharmacokinetics, and clinical efficacy are required. Third, the safety of *Ephedra* must also be examined. More attention should be paid to the acute, subacute, and long-term toxicity of *Ephedra* alkaloids and to the determination of the target organs suffering from toxicity; in vivo and clinical studies must be conducted to determine the range of safe doses of *Ephedra*, standardize its safe use, and expand the scope of clinical application. Fourth, as *Ephedra* is usually used together with other traditional Chinese medicines in the form of a prescription; however, the data on its active ingredients and mechanisms of action after combined use are still unclear. Finally, new research techniques and means are essential to promote the application of *Ephedra*. To summarize, we think that with increased research on traditional Chinese medicine, including *Ephedra*, we should first fully collect, accumulate, and study the data, so that our understanding of traditional Chinese medicine is accurate and reliable. On this basis, we should explore and summarize the application and efficacy of *Ephedra* or extend its application in other directions. Then, with a large amount of experimental data and repeated clinical verification, we can obtain relatively reliable conclusions. Notably, although we can try to find the specific indications of drugs by applying experimental methods of modern medicine, we cannot completely rely on them to guide clinical drug use, because the scope of drug dosage and the effect and mechanism of action for treating diseases are not yet clear enough. Therefore, the clinical use of drugs should not only be guided by practical experience, but should also be based on the main principle of combining traditional Chinese medicine theory with scientific research data. The drug application scope should be determined or expanded on the premise of ensuring safety; otherwise, the effectiveness and safety of drug use cannot be guaranteed.

In this study, through the review of many studies, we systematically summarized the clinical application of Ephedra for the first time. We collated a large number of toxicity studies on EHE and EFE components, and we and summarized the botany, chemical components, and pharmacological effects of *Ephedra*. We also described the future development direction of *Ephedra*.

## Figures and Tables

**Table 1:** Alkaloids in *Ephedra sinica* Stapf (structural formulas are shown in Figure S1).

No.	Classification	Compound Name	Ref.
1	Macrocyclic spermine alkaloids	Ephedradine A	[12]
2		Ephedradine B	[13]
3		Ephedradine C	[13]
4		Ephedradine D	[14]
5	Imidazole alkaloids	Feruloylhistamine	[15]
6	Amphetamine-type alkaloids	D(–)-Ephedrine	[16]
7		L(+)-Pseudoephedrine	[17] [18]
8		D(–)Norephedrine	[17] [18]
9		L(+)-Noreseudoephedrine	[17] [18]
10		D(–)Methylephedrine	[17] [18]
11		L(+)-Methylpseudoephedrine	[17] [18]
12		Ephedroxane	[19] [20]
13		3, 4-Dimethyl-5-pheyloxazolidine	[20]
14		2, 3, 4-Trimethyl-5-phenyloxazolidine	[20]
15		O-benzoyl-L(+)-pseudoephedrine	[20]
16		O-benzoyl-D(–)-ephedrine	[20]
17		Hordenine	[21]
18		(S)-N-((1R, 2S)-1-hydroxy-1-phenylpropan-2-yl)-5-oxopyrrolidine-2-carboxamide	[22]
19	Quinoline alkaloids	Transtorine	[23]
20		6-Methoxykynurenic acid	[24]
21		Kynurenic acid	[24]
22		6-Hydroxykynurenic acids	[24]
23		Ephedralone	[25] [26]
24	Pyrrolidine alkaloids Other alkaloids	cis-3, 4-Methanoproline	[27]
25		Maokonine	[28]
26		(±)-1-Phenyl-2-imido-1-propanol	[29]
27		Tetramethylpyrazine	[30]
28		Benzylamine	[31]
29		N-methybenzlamine	[32]

**Table 2:** Flavonoids in *Ephedra sinica* Stapf (structural formulas are shown in Figure S2).

No.	Classification	Compound Name	Ref.
31	Flavonols	Herbacetin	[36]
32		Kaempferol	[37]
33		Quercetin	[37]
34		Herbacetin 7-methylether	[36]
35		Rutin	[38]
36		Herbacetin 8-methyl ether3-*O*-glucoside-7-*O*-rutinoside	[39]
37		Herbacetin 7-*O*-(6″-quinylglucoside)	[39]
38		Herbacetin 3-*O*-rhamnoside 8-*O*-glucoside	[26]
39		Pollenitin B	[40]
40		Herbacetin-8-methyl ether 3-*O*-glucoside	[36]
41		Herbacetin 7-*O*-glucoside	[40]
42		Kaempferol 3-*O*-rhamnoside 7-*O*-glucoside	[40]
43		Herbacetin 7-*O*-neohesperidoside	[40]
44		Kaempferol-3-*O*-glucoside-7-*O*-rhamnoside	[40]
45		Kaempferol 3-*O*-rhamnoside	[39]
46		Quercetin 3-*O*-rhamnoside	[39]
47		Quercetin-3-*O*-glucoside	[38]
48	Dihydroflavonol	Dihydroquercetin	[37]
49		3-Hydroxynaringenin	[41]
50	Flavonone	3′, 4′, 5, 7-Tetrahydroxy flavanone	[37]
51		Naringenin	[41]
52		Hesperidin	[42]
53	Flavanols	(–)-epicatechin	[43]
54		(–)-epiafzelechin	[37]
55		Gallocatechin	[44]
56		Epigallocatechin	[37]
57		Leucoanthpcyanin	[45]
58		Catechin	[40]
59		Afzelechin	[37]
60		Leucocyanidin	[46]
61		Symplocoside	[40]
62	Flavones	Tricin	[36]
63		Luteolin	[47]
64		Luteolin-7-glucoside	[33]
65		Apigeni	[37]
66		3-Methoxyherbacetin	[36]
67		Apigenin-5-rhamnoside	[36]
68		6-C-glycosyl-chrysoeriol	[48]
69		Swertisin	[49]
70		Isovitexin	[50]
71		Isovitexin-2″-*O*-rhamnoside	[40]
72		Apigenin-7-*O*-glucoside	[38]
73		Vitexin	[40]
74		Lucenin III	[39]
75		2″, 2′″-Di-*O*-β-glucopyranosyl-vicenin II	[51]
76		6, 8-di-C-hexosyl apigenin	[44]
77		6/8-C-hexosyl-8/6-C-pentasyl apigenin	[44]
78	Anthocyan	Leucodelphinidin	[46]
79		Leucopelargonin	[46]

**Table 3:** Tannins in *Ephedra sinica* Stapf (structural formulas are shown in Figure S3).

No.	Classification	Compound Name	Ref.
80	Dimer proanthocyanidins	Ephedrannin A	[53]
81		Ephedrannin B	[53]
82		Muhuannin A	[54]
83		Muhuannin D	[53]
84		Muhuannin B	[53]
85		Muhuannin E	[53]
86		Muhuannin C	[38]
87		Muhuannin F	[55]
88		Muhuannin G	[55]
89		Muhuannin H	[56]
90		Muhuannin I	[55]
91		Muhuannin J	[56]
92		Muhuannin K	[55]
93		Ephedrannin D1	[44]
94		Ephedrannin D2	[44]
95		Ephedrannin D3	[40]
96		Ephedrannin D4	[44]
97		Ephedrannin D5	[44]
98		Ephedrannin D6	[44]
99		Ephedrannin D7	[44]
100		Ephedrannin D8	[44]
101		Ephedrannin D9	[44]
102		Ephedrannin D10	[44]
103		Ephedrannin D11	[44]
104		Ephedrannin D12	[44]
105		Ephedrannin D13	[44]
106		Ephedrannin D14	[44]
107	Trimer proanthocyanidins	Ephedrannin Tr1	[44]
108		Ephedrannin Tr2	[44]
109		Ephedrannin Tr3	[44]
110		Ephedrannin Tr4	[44]
111		Ephedrannin Tr5	[44]
112		Ephedrannin Tr6	[44]
113		Ephedrannin Tr7	[44]
114		Ephedrannin Tr8	[44]
115		Ephedrannin Tr9	[44]
116		Ephedrannin Tr10	[44]
117		Ephedrannin Tr11	[44]
118		Ephedrannin Tr12	[44]
119		Ephedrannin Tr13	[44]
120		Ephedrannin Tr14	[40]
121		Ephedrannin Tr15	[40]
122	Tetramer proanthocyanidins	Ephedrannin Te1	[40]
123		Ephedrannin Te2	[40]
124		Ephedrannin Te3	[40]
125		Ephedrannin Te4	[40]
126		Ephedrannin Te5	[40]
127	Hydrolytic tannins	Nilocitin	[39]

**Table 4:** Organic acids in *Ephedra sinica* Stapf (structural formulas are shown in Figure S4).

No.	Classification	Compound Name	Ref.
128	Organic acids	Nebrodenside A	[22] [43]
129		Nebrodenside B	[22] [43]
130		*O*-coumaric acid glucoside	[22] [58]
131		Trans-cinnamic acid	[58]
132		Syringin	[45]
133		*O*-Coumaric acid	[25]
134		ρ-Hydroxybenzoic acid	[56]
135		Protocatechuic acid	[58]
136		Quinaldic acid	[59]
137		2-Hydroxyl-5-methoxybenzoic acid	[55]
138		Iso-ferulic acid	[55]
139		Vanillic acid	[56]
140		Caffeic acid	[58]
141		Chlorogenic acid	[58]
142		(3R)-3-*O*-β-D-glucopyranosyl-3-phenylpropanoic acid	[22]
143		Malic acid	[60]
144		Citric acid	[60]
145		Oxalic acid	[60]
146		Fumaric acid	[60]
147		4-*O*-β-D-glucoside benzoic acid	[61]
148		5-(hydroxy-isopropyl)-cyclohexenecarboxylic acid	[58]
149		Pseudolaroside B	[61]
150		n-hexacosane acid	[62]
151		Trans-aconitic acid	[50]

**Table 5:** Organic volatile essential oil in *Ephedra sinica* Stapf (structural formulas are shown in Figure S5).

No.	Classification	Compound Name	Ref.
152	Organic volatile essential oil	β-Sitosterol	[63]
153		9Z, 12Z-Octadecadienoic acid	[63]
154		9-E-Octadecenoic acid	[63]
155		Ergost-5-en-3β-ol	[63]
156		Nonacosanol	[63]
157		L-α-terpineol	[69]
158		Linolenic acid	[63]
159		Terpineol acetate	[70]
160		3, 7, 11, 15-Tetramethyl-2-hexadecen-1-ol	[63]
161		Stearic acid	[63]
162		Globulol	[70]
163		γ-eudesmol	[69]
164		Linalool	[69]
165		Eicosanoic acid	[63]
166		Cis-2-ρ-menthen-7-ol	[69]
167		Terpinen-4-ol	[69]
168		β-terpineol	[71]
169		Myrcene	[71]
170		Dihydrocarveol	[71]
171		1, 3, 4-Trimethyl-3-cyclohexene-1-carboxaldehyde	[71]
172		Trans-phytol	[72]
173		Linolenic acid methyl ester	[72]
174		γ-Sitosterol	[72]
175		1, 4-Cineole	[69]
176		1, 8-Cineole	[69]
177		ρ-Cymene	[69]
178		Limonene	[69]
179		γ-Terpinene	[69]
180		Hexadecanoic acid	[69]
181		Dibutyl phthalate	[69]

**Table 6:** Other components in *Ephedra sinica* Stapf (structural formulas are shown in Figure S6).

No.	Classification	Compound Name	Ref.
182	Lignans	DL-Syringaresinol	[25]
183		Sesquipinsapol B	[55]
184	Naphthalenes	Methyl-2,3-methylenedioxy-6-naphthalenecarboxylic acid methyl ester	[58]
185	Esters	Ethyl caprylate	[73]
186	Terpenoids	(–)-α-Terpineol-8-*O*-β-D-glucopyranoside	[55]
187		(+)-α-Terpineol-8-*O*-β-D-glucopyranoside	[55]
188		Geranyl-β-D-glucopyranoside	[55]
189		Daucosterol	[55]
190		Sitosterol	[55]
191		Stigmasterol-3-*O*-β-D-glucopyranoside	[73]
192	Quinones	Physcion	[58]
193		Rhein	[58]
194	Phenols	ρ-Aminophenol	[58]
195		Rhododendrol-4′-*O*-β-D-glucopyranoside	[61]
196		Vinylguaiacol	[72]
197		Di-tert-butylphenol	[72]
198		Antiarol	[72]
199	Ureas	Allantoin	[44]

## Data Availability

Not applicable.

## Supplementary Materials

The following supporting information can be downloaded at: https://www.mdpi.com/article/10.3390/molecules28020663/s1. Figure S1: Alkaloids isolated from *Ephedra sinica* Stapf. Figure S2: Flavonoids isolated from *Ephedra sinica* Stapf. Figure S3: Tannins isolated from *Ephedra sinica* Stapf. Figure S4: Organic phenolic acids isolated from *Ephedra sinica* Stapf. Figure S5: Organic volatile essential oils isolated from *Ephedra sinica* Stapf. Figure S6: Other ingredients isolated from *Ephedra sinica* Stapf.

## Abbreviations

*Ephedra*, *Ephedra sinica* Stapf; COVID-19, novel coronavirus pneumonia 2019; TFC, total flavonoid content; β2-AR, 2-adrenoceptor; OVA, ovalbumin; ELISA, enzyme-linked immunosorbent assay; G+, Gram-positive bacteria; PGN, peptidoglycan; TLR2, Toll-like receptor 2; DCs, dendritic cells; HPLC-PDA, high-performance liquid chromatography with photodiode array detector; SH, Suhuangzhenke capsule; D-GalN/LPS, D-galactosamine–lipopolysaccharide; CCl4, carbon tetrachloride; DPPH, 2, 2′-diphenyl-1-pyridohydrazine; SOD, superoxide dismutase; HFD, high-fat diet; NAFLD, nonalcoholic fatty liver disease; ROS, reactive oxygen species; ALT, alanine aminotransferase; T.Bil, total bilirubin; AFB(1), aflatoxin B(1); HGF, hepatocyte growth factor; NF-κB, nuclear factor-κB; HIV-1, human immunodeficiency virus type 1; IFV, influenza virus; QFPDT, Qingfei Paidu decoction; ASA, acetylsalicylic acid; PSE, pseudoephedrine; c-Met, cellular mesenchymal-to-epithelial transition factor; NOAEL, no observable adverse effect level; EHE, *Ephedra* extract; EFE, ephedrine alkaloid-free *Ephedra* Herb extract; TRPV1, transient receptor potential vanilloid 1; TCM, traditional Chinese medicine; bpm, beat per minute.
