# Supplementary material for: Ephedrae Herba: A Review of Its Phytochemistry, Pharmacology, Clinical Application, and Alkaloid Toxicity
Source: Molecules. 2023 Jan 9;28(2):663. doi: 10.3390/molecules28020663 (PMC9863261; doi:10.3390/molecules28020663)
Supplement: Supplementary file 1 [file molecules-28-00663-s001.zip › molecules-2069718-SI.pdf]

Supplementary Material

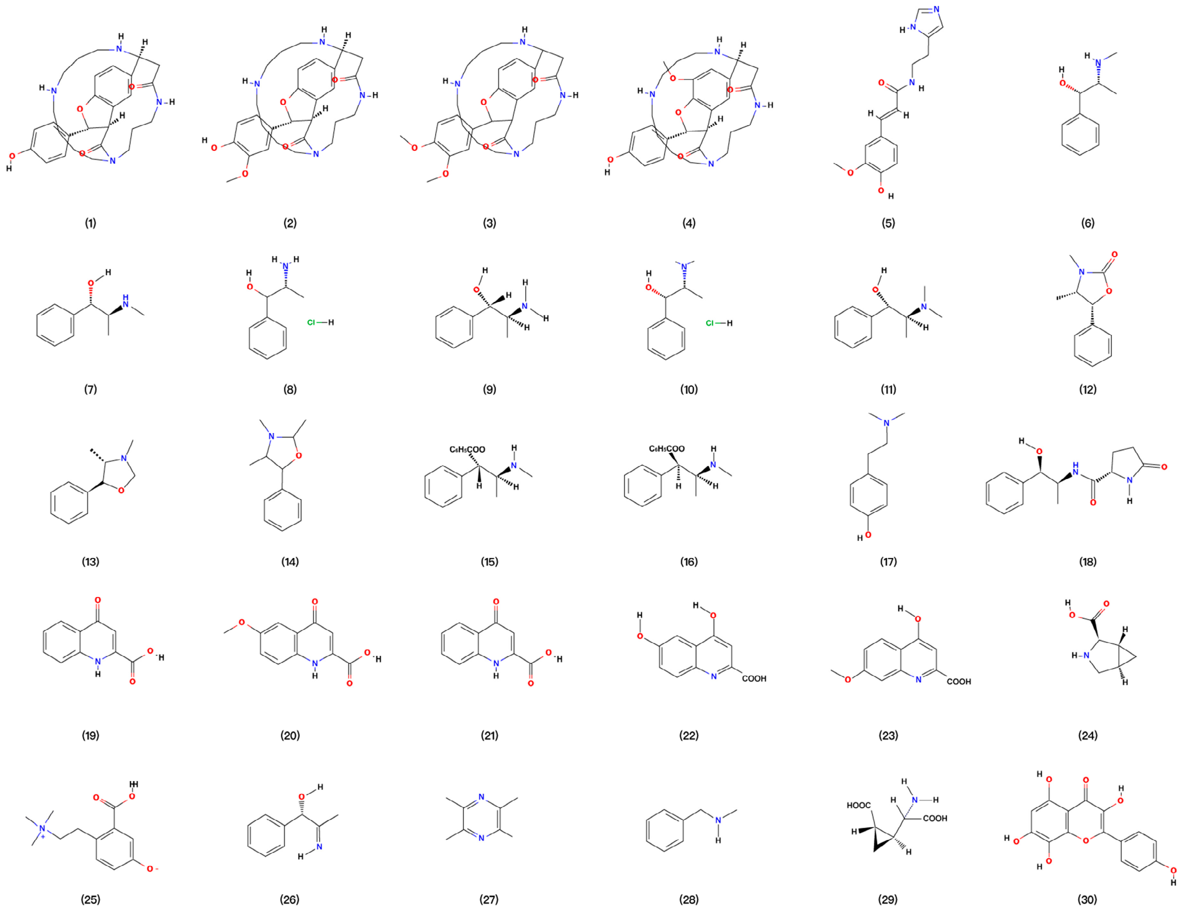

Figure S1. Alkaloids isolated from *Ephedra sinica* Stapf.

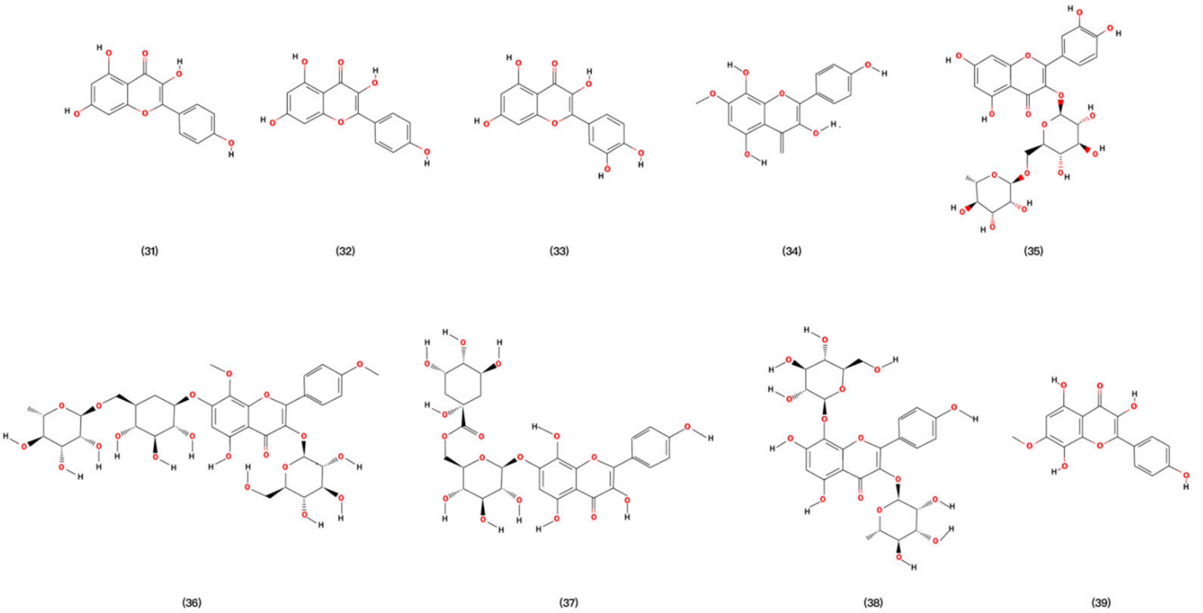

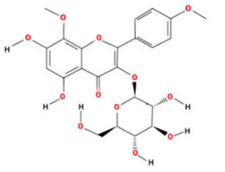

(40)

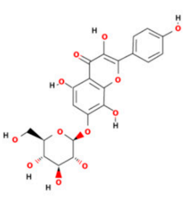

(41)

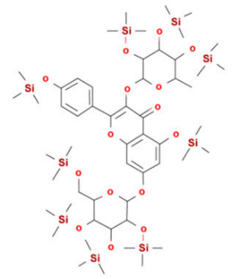

(42)

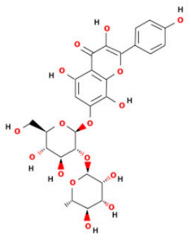

(43)

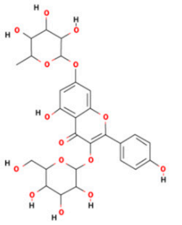

(44)

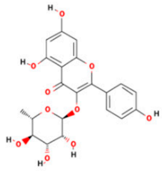

(45)

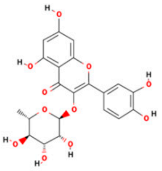

(46)

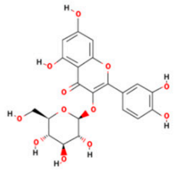

(47)

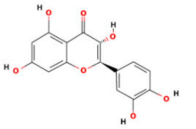

(48)

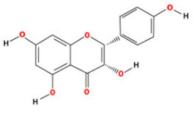

(49)

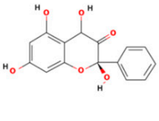

(50)

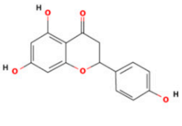

(51)

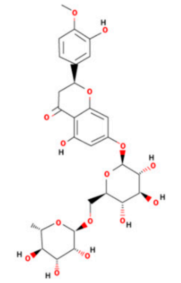

(52)

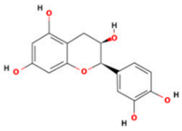

(53)

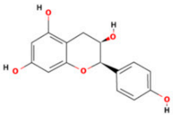

(54)

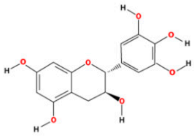

(55)

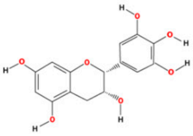

(56)

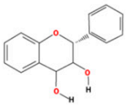

(57)

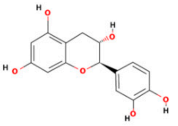

(58)

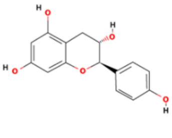

(59)

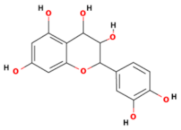

(60)

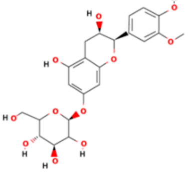

(61)

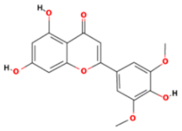

(62)

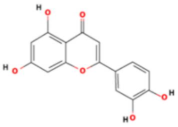

(63)

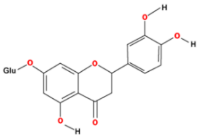

(64)

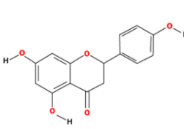

(65)

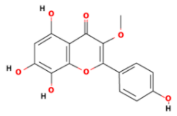

(66)

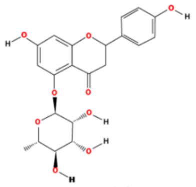

(67)

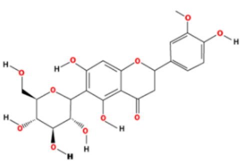

(68)

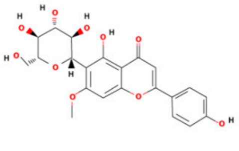
(69)

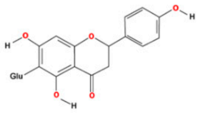
(70)

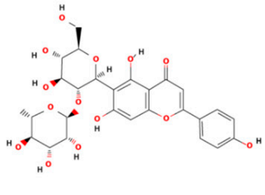
(71)

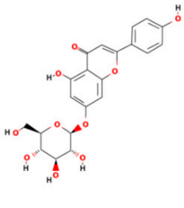
(72)

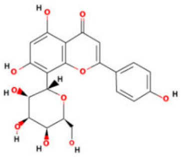
(73)

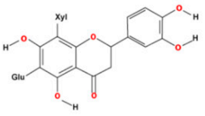
(74)

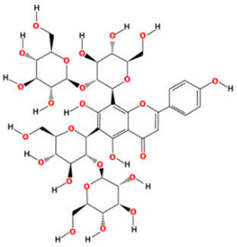
(75)

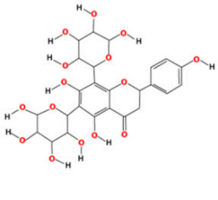
(76)

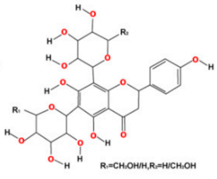

(77)

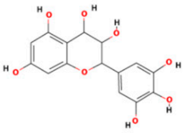
(78)

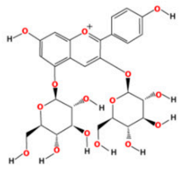
(79)

Figure S2. Flavonoids isolated from *Ephedra sinica* Stapf.

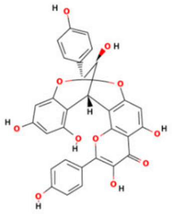
(80)

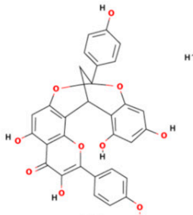
(81)

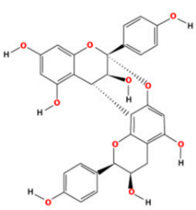
(82)

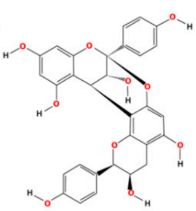
(83)

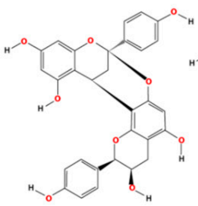
(84)

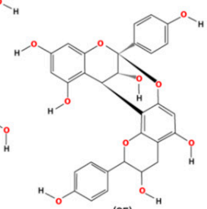
(85)

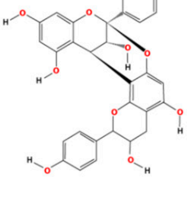
(86)

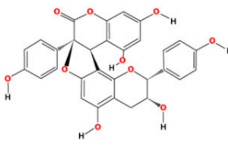
(87)

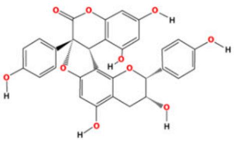
(88)

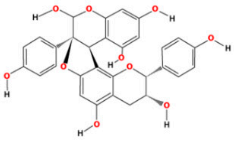
(89)

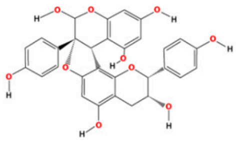
(90)

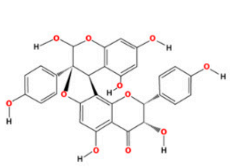

(91)

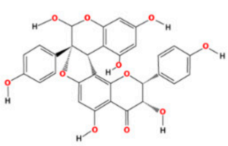

(92)

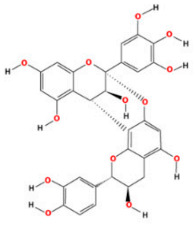

(93)

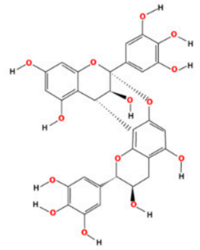

(94)

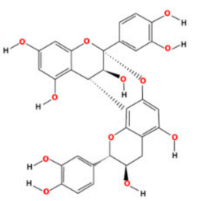

(95)

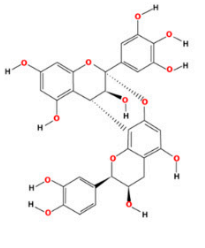

(96)

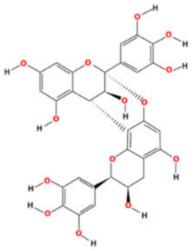

(97)

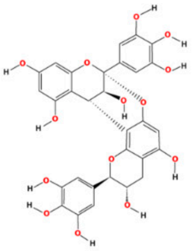

(98)

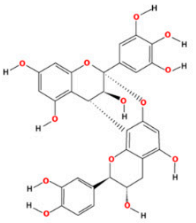

(99)

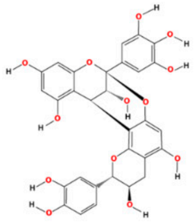

(100)

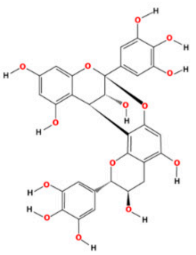

(101)

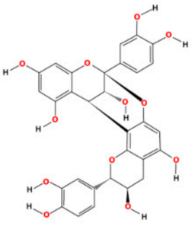

(102)

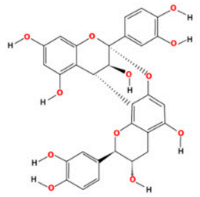

(103)

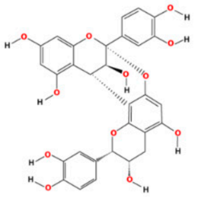

(104)

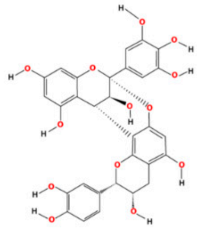

(105)

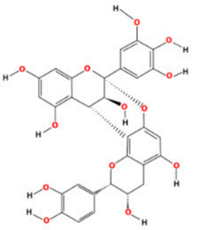

(106)

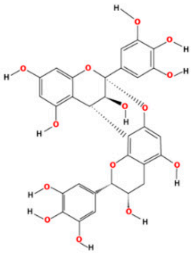

(107)

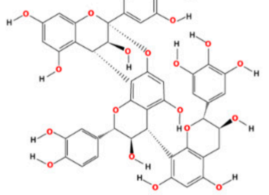

(108)

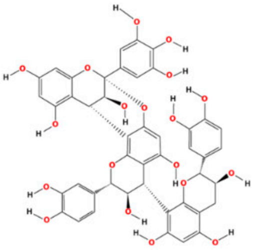

(109)

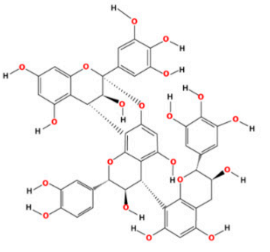

(110)

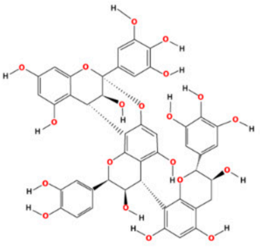

(111)

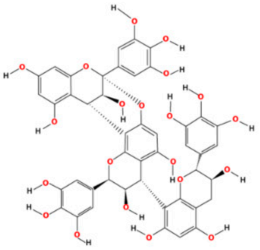

(112)

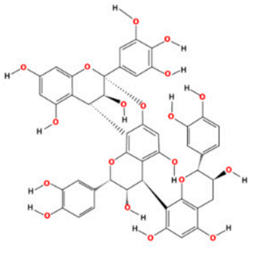

(113)

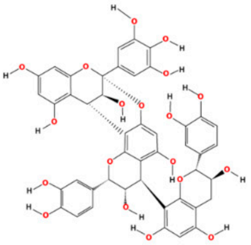

(114)

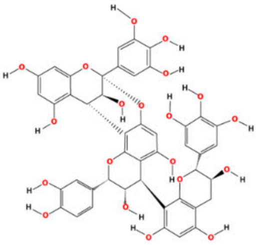

(115)

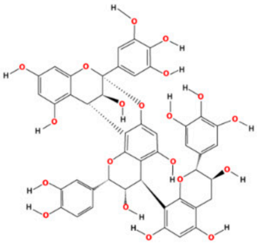

(116)

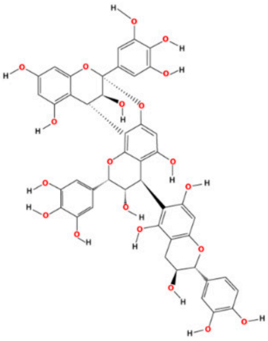

(117)

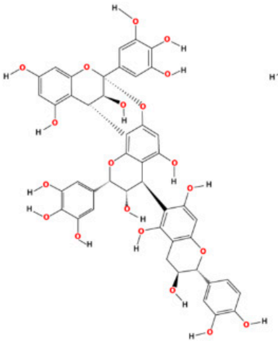

(118)

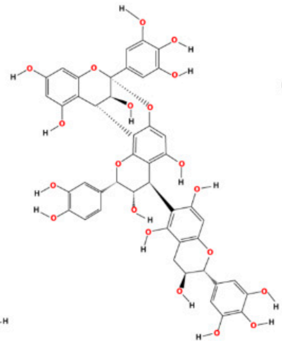

(119)

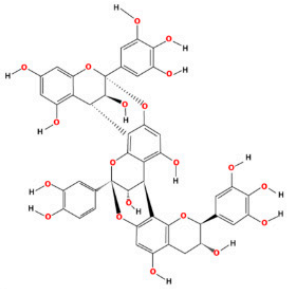

(120)

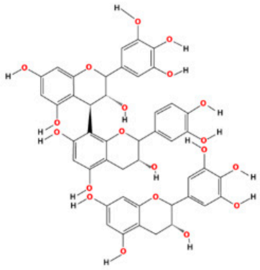

(121)

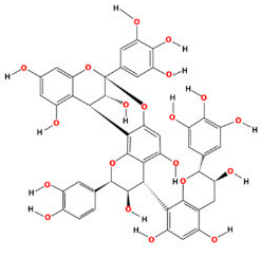

(122)

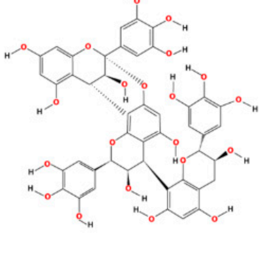

(123)

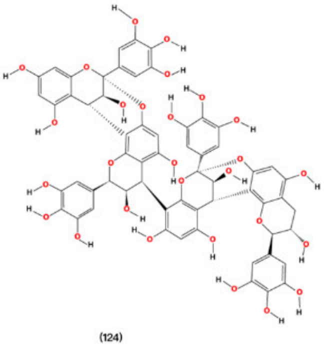

(124)

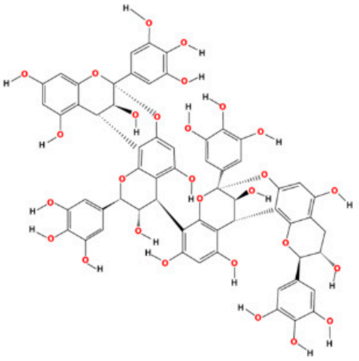

(125)

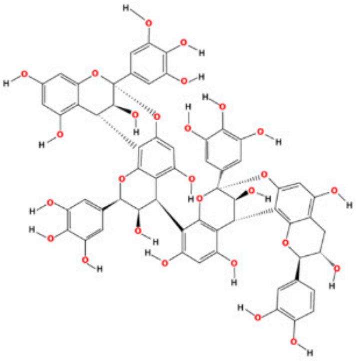

(126)

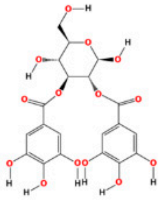

(127)

Figure S3. Tannins isolated from *Ephedra sinica* Stapf.

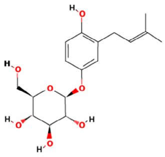

(128)

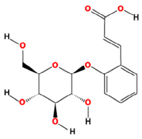

(129)

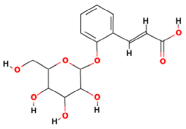

(130)

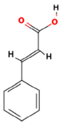

(131)

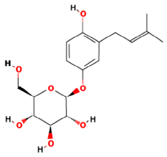

(132)

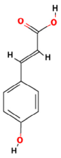

(133)

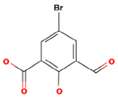

(134)

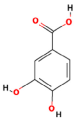

(135)

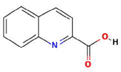

(136)

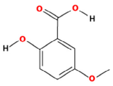

(137)

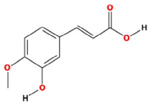

(138)

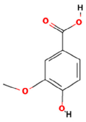

(139)

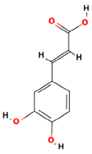

(140)

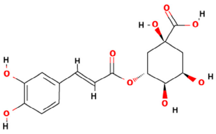

(141)

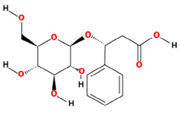

(142)

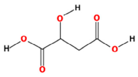

(143)

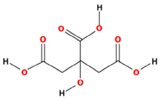

(144)

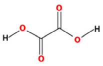

(145)

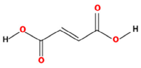

(146)

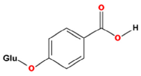

(147)

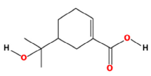

(148)

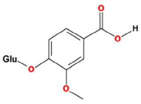

(149)

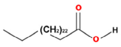

(150)

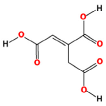

(151)

Figure S4. Organic phenolic acids isolated from *Ephedra sinica* Stapf.

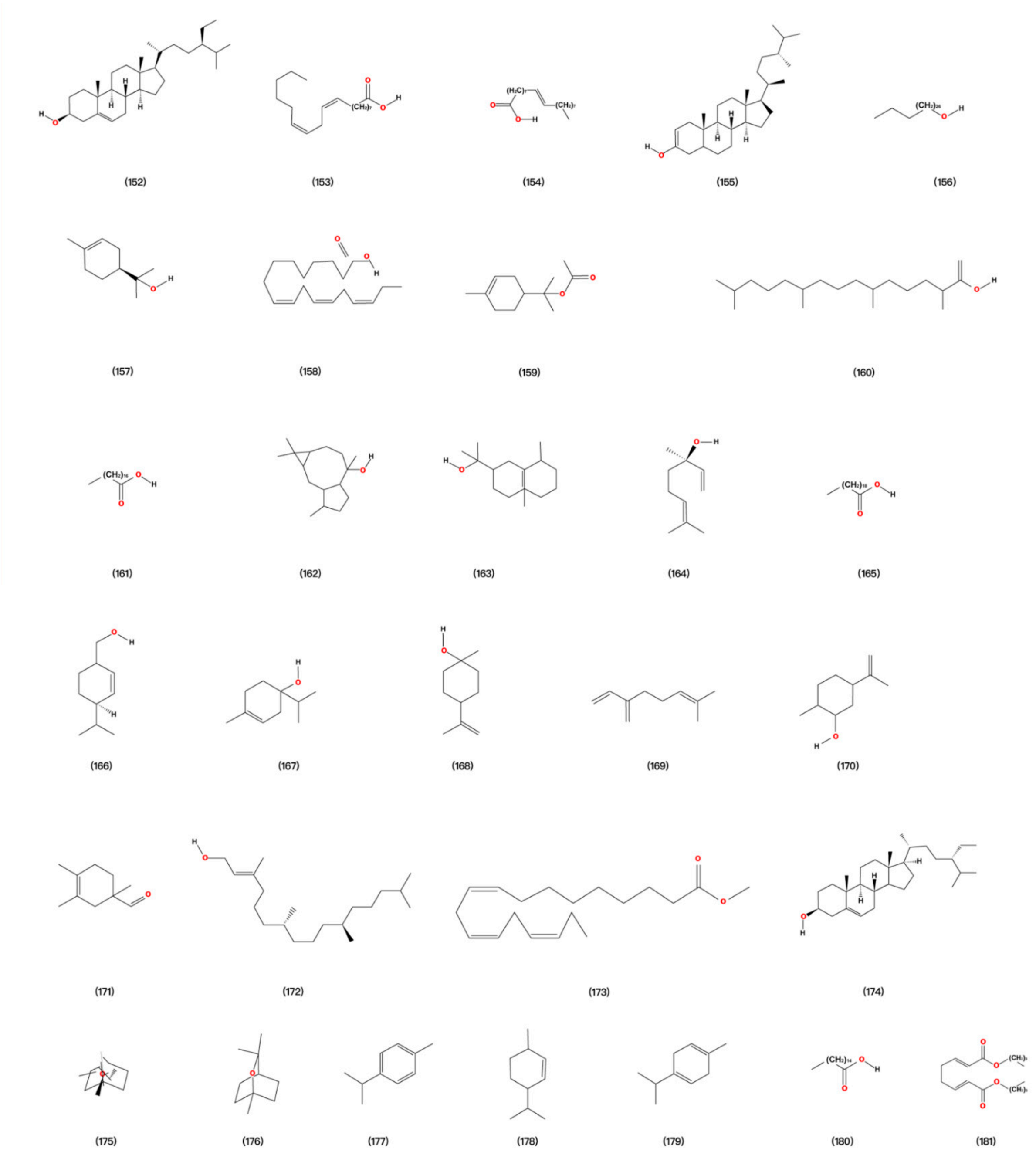

Figure S5. Organic volatile essential oils isolated from *Ephedra sinica* Stapf.

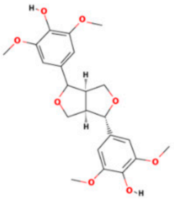

(182)

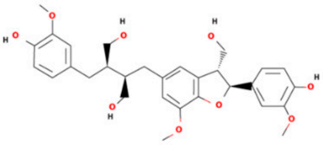

(183)

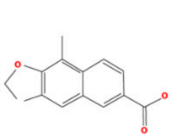

(184)

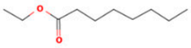

(185)

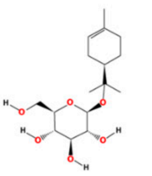

(186)

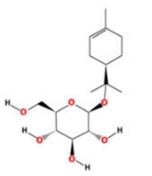

(187)

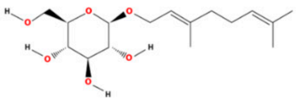

(188)

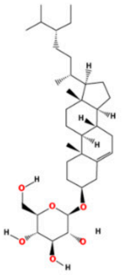

(189)

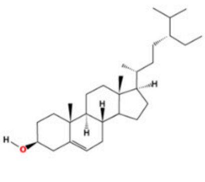

(190)

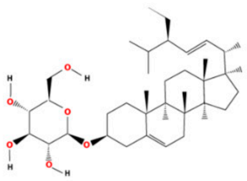

(191)

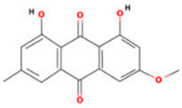

(192)

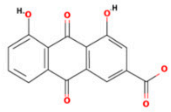

(193)

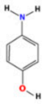

(194)

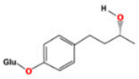

(195)

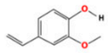

(196)

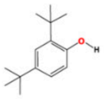

(197)

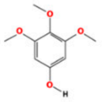

(198)

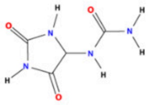

(199)

Figure S6. Other ingredients isolated from *Ephedra sinica* Stapf.
